# Solution Structure of MSL2 CXC Domain Reveals an Unusual Zn_3_Cys_9_ Cluster and Similarity to Pre-SET Domains of Histone Lysine Methyltransferases

**DOI:** 10.1371/journal.pone.0045437

**Published:** 2012-09-20

**Authors:** Sanduo Zheng, Jia Wang, Yingang Feng, Jinfeng Wang, Keqiong Ye

**Affiliations:** 1 Department of Biochemistry and Molecular Biology, College of Life Sciences, Beijing Normal University, Beijing, China; 2 National Institute of Biological Sciences, Beijing, China; 3 Shandong Provincial Key Laboratory of Energy Genetics, Qingdao Institute of BioEnergy and Bioprocess Technology, Chinese Academy of Sciences, Qingdao, Shangdong, China; 4 National Laboratory of Biomacromolecules, Institute of Biophysics, Chinese Academy of Sciences, Beijing, China; George Washington University, United States of America

## Abstract

The dosage compensation complex (DCC) binds to single X chromosomes in *Drosophila* males and increases the transcription level of X-linked genes by approximately twofold. Male-specific lethal 2 (MSL2) together with MSL1 mediates the initial recruitment of the DCC to high-affinity sites in the X chromosome. MSL2 contains a DNA-binding cysteine-rich CXC domain that is important for X targeting. In this study, we determined the solution structure of MSL2 CXC domain by NMR spectroscopy. We identified three zinc ions in the CXC domain and determined the metal-to-cysteine connectivities from ^1^H-^113^Cd correlation experiments. The structure reveals an unusual zinc-cysteine cluster composed of three zinc ions coordinated by six terminal and three bridging cysteines. The CXC domain exhibits unexpected structural homology to pre-SET motifs of histone lysine methyltransferases, expanding the distribution and structural diversity of the CXC domain superfamily. Our findings provide novel structural insight into the evolution and function of CXC domains.

## Introduction

Organisms with different numbers of sex chromosomes between males and females face the problem of an unequal dosage of genes from sex chromosomes. In *Drosophila melanogaster*, the transcriptional level of most genes in the single male X chromosome is increased by approximately twofold to match that from two female X chromosomes (see recent reviews [1]–[3]). This dosage compensation process is mediated by the dosage compensation complex (DCC) or male-specific lethal (MSL) complex, which contains at least five proteins MSL1, MSL2, MSL3, males absent on the first (MOF) and maleless (MLE) and two non-coding RNAs roX1 and roX2. MSL1 is a scaffold protein associated with MSL2, MSL3 and MOF [4]–[6].

In male flies, the DCC is located at hundreds of sites along the length of the X chromosome. Each of five proteins and at least one of the functionally redundant roX RNAs are required for full association of the DCC on the X chromosome and for male viability. The DCC is not assembled in females because MSL2 translation is tightly repressed [7], [8]. The DCC has been shown to primarily bind at bodies of active genes on the X chromosome [9], [10]. The transcriptional activation is caused, at least in part, by the MOF-mediated acetylation of histone H4 lysine 16 and enhanced transcriptional elongation [11].

The mechanism by which the DCC is specifically localized to the X chromosome remains poorly understood. According to a prevalent model, the DCC first binds to a limited number of high-affinity sites (HAS) or chromatin entry sites (CES) in the X chromosome and then spreads *in cis* to flanking active genes [12]. The spreading process probably involves the interaction of the MSL3 chromodomain with trimethylated H3K36, a marker for actively transcribed genes [13]. HAS are able to attract even a partially assembled DCC that lacks MSL3, MOF or MLE, or a low concentration of DCC [7], [14], [15]. A body of evidence suggests that specific DNA sequences are involved in HAS recognition. When translocated to an autosome, HAS as short as 100–200 base pairs (bp) can still recruit the DCC [16]–[20]. Chromatin immunoprecipitation studies followed by microarray analysis or deep sequencing have mapped ∼140 HAS on the X chromosome [20], [21]. The binding sites of the DCC on HAS are enriched with a GA-rich MSL recognition element (MRE) [20]. However, the MRE motif occurs frequently outside of HAS and is only slightly enriched in the X chromosome over autosomes, indicating that the MRE motif is not the sole determinant for HAS recognition. The conformation of chromatin also appears to be important for HAS recognition, since HAS are characterized by low nucleosome occupancy [20] and special compartments in the nuclei [22].

MSL2 is a core component of the DCC [23]–[25] and together with MSL1 is required for the DCC to bind HAS on the X [14], [15], [26], [27]. MSL2 was recently shown to be a DNA-binding protein and specifically recognize a HAS in reporter gene assay in *Drosophila* cells [28]. However, MSL2 failed to discriminate the HAS sequence in vitro. An unknown selectivity cofactor was proposed to cooperate with MSL2 in vivo for specific HAS recognition [28].

MSL2 is composed of an N-terminal RING domain, a cysteine-rich CXC domain and a C-terminal region rich in proline and basic residues (Pro/Bas patch). The RING domain binds MSL1 [6], [29] and exhibits ubiquitin E3 ligase activity toward H2B K34 [30]. The CXC domain contributes critically to the DNA-binding activity of MSL2 [28]. CXC domains are also present, mostly in two copies, in the tesmin/TSO1 protein family [31]–[35]. The tandem CXC domain of human LIN54 and soybean CPP1 has been shown to bind specific DNA sequences [32], [33]. No structure has been reported for any CXC domain.

The CXC domain is remarkable by having 9 invariant Cys within about 50 residue region. In this study, we have determined the first structure of MSL2 CXC domain by NMR spectroscopy. The structure reveals a compact fold that encages an unusual Zn_3_Cys_9_ cluster. Interestingly, the CXC structure with a Zn_3_Cys_9_ cluster shows strong similarity to pre-SET motifs of histone lysine methyltransferases, suggesting that the CXC and pre-SET domains share a common evolutionary origin.

## Results

### The MSL2 CXC Domain is an Autonomously Folded Structure Containing Three Zinc Ions

**Figure 1:**
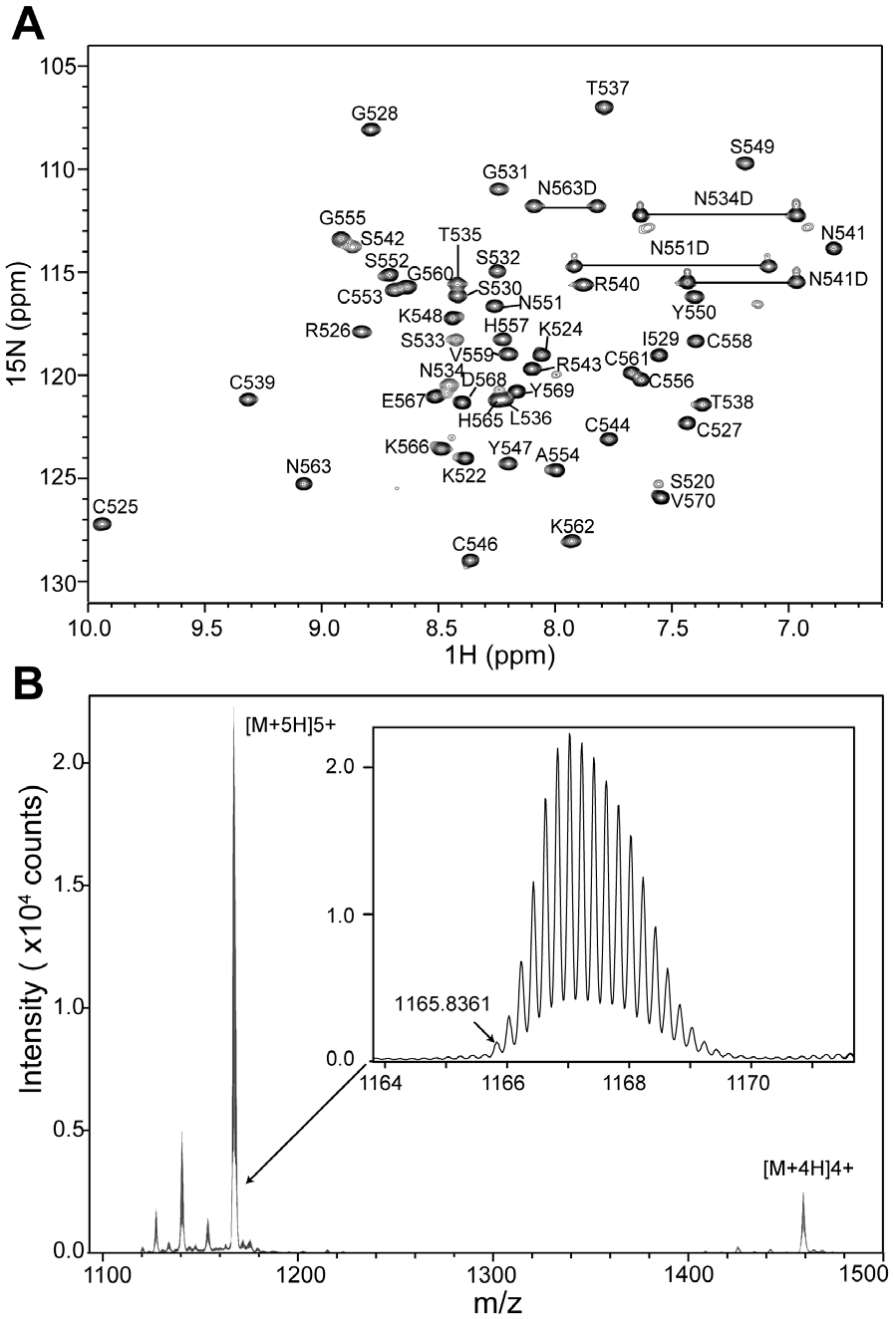
The CXC domain of MSL2 binds three zinc ions. (A) ^1^H-^15^N HSQC spectrum of the CXC domain. The spectrum was collected on 1 mM ^15^N-labeled CXC-3 protein in 50 mM phosphate buffer (pH 6.0) and 10% ^2^H_2_O at 25°C and 600 MHz. The assigned residues are labeled with full-length MSL2 numbering, and the side-chain amide protons are connected by lines. (B) Electrospray ionization mass spectroscopy reveals three bound Zn^2+^ ions. The CXC-3 protein was exchanged into 200 mM ammonia acetate and analyzed with a Q-Star instrument. The inset shows the major isotopic peak series from a +5 charge species. The monoisotopic peak (m/z 1165.8361) corresponds to an exact mass of 5824.1805 Da, consistent with a complex of CXC-3 and three Zn^2+^ ions (monoisotopic MW = 5824.267 Da). The peak series around m/z = 1460 originates from a +4 charge species.

Our structural analysis of *D. melanogaster* MSL2 CXC domain was conducted mainly on two constructs. One construct containing residues 517–572 and the C560G mutation (CXC-2) was used in early experiments, and another slightly shorter construct containing residues 520–570 and the C560G mutation (CXC-3) was used in the majority of experiments. The two fragments display identical NMR spectra in folded regions and thus should have the same structure. In both constructs, the nonconserved residue Cys560 was replaced by glycine, an amino acid that is frequently found at the corresponding position in MLS2 homologs, to avoid formation of intermolecular disulfide bonds. The ^1^H-^15^N HSQC spectrum of the MSL2 CXC domain displays a single set of well-dispersed peaks, indicating that the fragment is autonomously folded and amenable for further structural characterization (Fig. 1A). Nearly complete assignments for backbone and side-chain resonances were obtained by analysis of a set of triple-resonance spectra collected on ^13^C/^15^N-labeled CXC protein.

The CXC domain is characterized by nine invariant cysteine residues and conserved spacing between them, suggesting that these cysteine residues may coordinate metal ions. Indeed, inductively coupled plasma mass spectrometry of recombinant CXC protein revealed a significant enrichment of Zn compared with other metals Fe, Mg, Ca, Mn, Ni and Cu (data not shown). To assess the Zn binding stoichiometry, the CXC-3 protein was analyzed with electrospray mass spectroscopy under conditions that preserve the native protein structure (Fig. 1B). The monoisotopic mass of the major species (5824.0875 Da) precisely matched that of a monomeric CXC-3 molecule in complex with three Zn^2+^ ions (5824.267 Da). Analytical ultracentrifuge sedimentation velocity assay further showed that the CXC domain is a monomer in solution (data not shown). These biophysical results indicate that the CXC domain folds into a monomeric structure with three bound zinc ions.

### Assignment of Zn-coordinating Ligands by ^113^Cd NMR

Three Zn^2+^ ions are most likely coordinated by nine invariant Cys residues in the MLS2 CXC domain. The CXC domain has two histidines at positions 557 and 565, but they are not conserved and are unlikely to bind structurally important Zn^2+^ ions. As each Zn^2+^ ion needs to be coordinated to four ligands, the unusual Zn to Cys ratio of 3∶9 suggests that these Zn^2+^ ions are bound in an unconventional way. To assign the zinc ligands, we replaced Zn with ^113^Cd, which has a similar coordination pattern to Zn but has more favorable NMR properties [36]. The ^113^Cd-loaded protein was prepared during protein expression by substituting Zn^2+^ with ^113^Cd^2+^ in minimal M9 medium.

**Figure 2:**
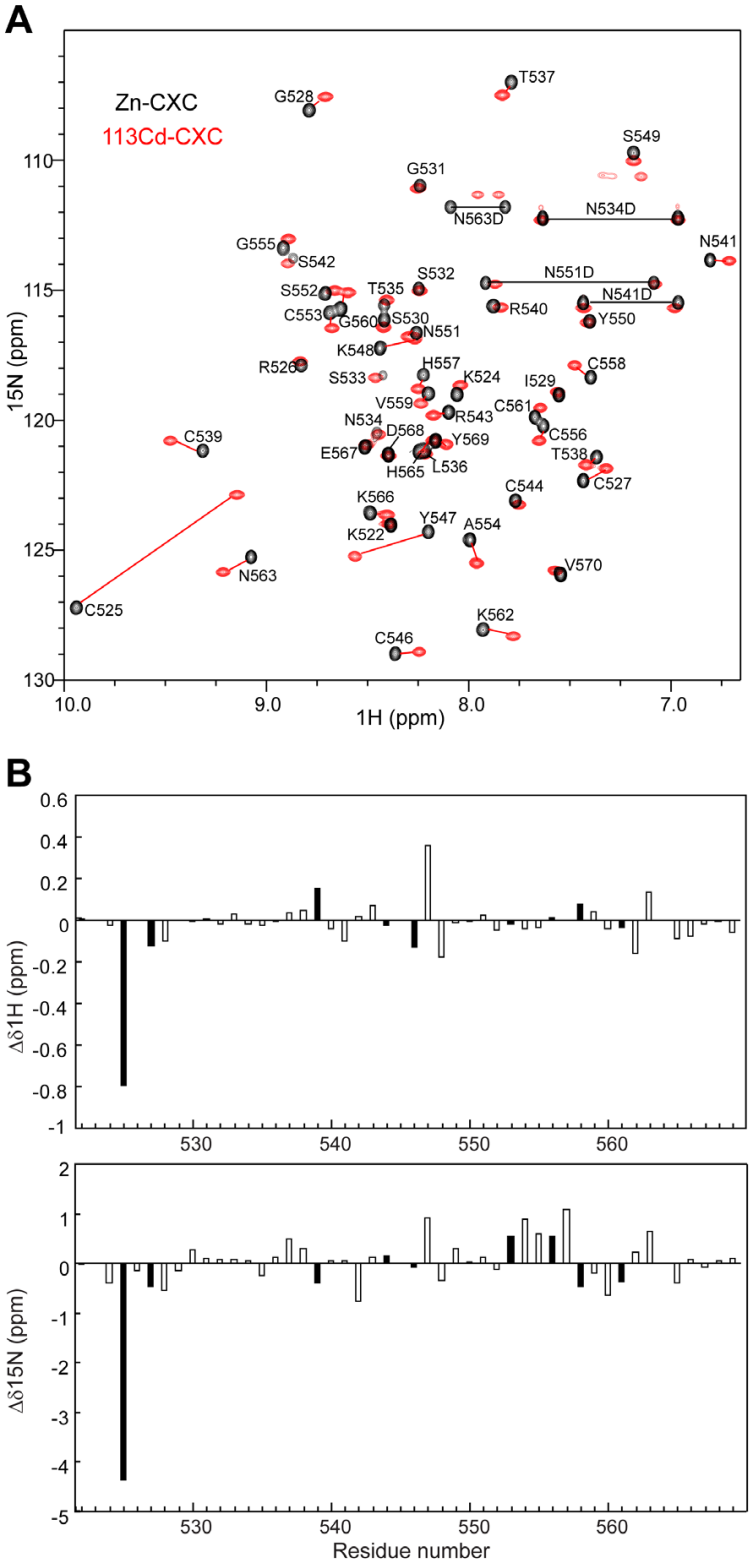
Chemical shift perturbation by ^113^Cd substitution. (A) Superimposed ^1^H-^15^N HSQC spectra of Zn-loaded (black) and ^113^Cd-loaded (red) CXC-3 proteins. Assignments are indicated for Zn-CXC. The distantly separated peak pairs are connected by red lines. The ^113^Cd-CXC assignments were confirmed with 3D ^1^H-^15^N TOCSY-HSQC and 3D ^1^H-^15^N NOESY-HSQC spectra collected on ^113^Cd/^15^N-labeled protein. (B) Chemical shift changes of the amide protons and nitrogens between ^113^Cd- and Zn-loaded CXC-3 proteins. Cys and non-Cys residues are shown with filled and empty bars. Δδ^1^H = δ^1^H_Cd-CXC_-δ^1^H_Zn-CXC_, Δδ^15^N = δ^15^N_Cd-CXC_-δ^15^N_Zn-CXC_.

The ^1^H-^15^N HSQC spectrum of ^113^Cd/^15^N-labeled CXC protein displays a single set of peaks, indicating complete ^113^Cd substitution (Fig. 2A). As many resonances were shifted after ^113^Cd replacement, the resonances of H, N, Hα and Hβ were reassigned using 3D ^1^H-^15^N TOCSY-HSQC and 3D ^1^H-^15^N NOESY-HSQC spectra collected on ^113^Cd/^15^N-labeled protein. The amide resonances of Cys residues and nearby residues generally display large changes upon ^113^Cd replacement (Fig. 2B). The chemical shifts of Hα and Hβ protons are less affected, with an average deviation of 0.048 ppm and a maximal deviation of <0.38 ppm. The NOE patterns are unaltered, indicating that the structure is minimally disturbed by ^113^Cd substitution.

**Figure 3:**
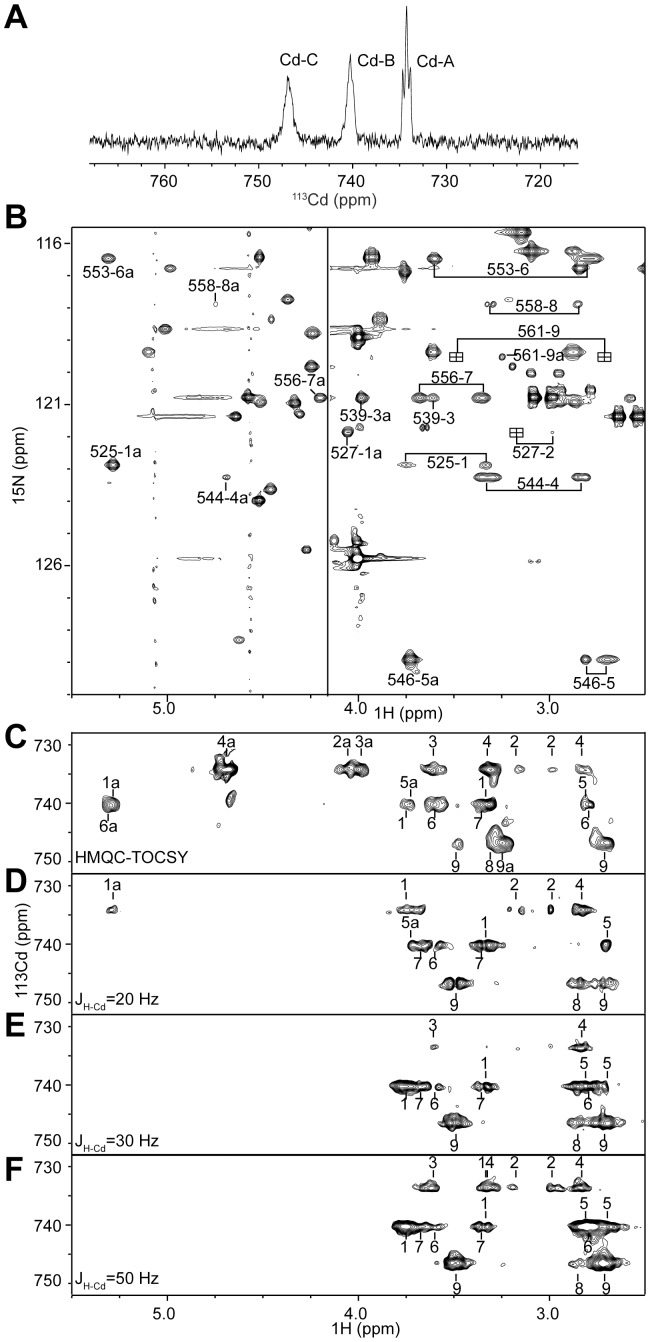
NMR spectra of ^113^Cd/^15^N-labeled CXC-3 protein. (A) 1D ^1^H-decoupled ^113^Cd spectrum. (B) 2D ^1^H-^15^N HSQC-TOCSY spectrum. The Cys residues at positions 525, 527, 539, 544, 546, 553, 556, 558 and 561 are sequentially numbered from 1 to 9. Cross-peaks originating from Cys Hβ are labeled with the residue number and dash-separated sequential number of Cys, and cross-peaks from Cys Hα are additionally marked with “a”. The spectrum is divided by a vertical line into two parts. The left Hα region is shown with a higher contour level than the right Hβ region for clarity. Some peaks from Cys527 and Cys561 marked by squares are not visible, and their positions were inferred from a 2D ^1^H-^15^N HSQC-NOESY spectrum. (C) ^1^H-^113^Cd HMQC-TOCSY spectra with^ 3^J_HB-Cd_ set to 50 Hz and a mixing time of 30 ms. (D-F) ^1^H-^113^Cd HSQC spectra with ^3^J_HB-Cd_ set to 20 Hz (D), 30 Hz (E) and 50 Hz (F). Assigned cross-peaks are marked with sequential number of Cys and those peaks from Hα are additionally labeled with “a”.

The ^1^H-decoupled 1D ^113^Cd spectrum shows three peaks at 734.2, 740.2 and 746.6 ppm (Fig. 3A), in agreement with three Zn^2+^ ions identified by native electrospray mass spectroscopy. These peaks appear as triplets but are most obvious for Cd-A. Each ^113^Cd ion is probably coupled to the other two ^113^Cd ions by sharing a bridging cysteine ligand, and the ^113^Cd-^113^Cd two-bond couplings (^2^J_Cd-Cd_ ∼30 Hz) split these resonances into triplets.

We collected a series of 2D ^1^H-^113^Cd HSQC spectra and an HMQC-TOCSY spectrum to correlate ^113^Cd ions to Hβ and Hα protons of coordinating Cys residues (Fig. 3C-F). Some Cys Hβ protons have close chemical shift values, hindering assignment of ^113^Cd coordination. To measure ^1^H chemical shifts with higher precision, we collected a 2D ^1^H-^15^N HSQC-TOCSY spectrum, in which ^1^H resonances were recorded in the more resolved direct dimension and could be directly aligned to ^1^H-^113^Cd HSQC spectra (Fig. 3B). Analysis of these spectra revealed that Cd-A (734.2 ppm) is coordinated to Cys525, Cys527, Cys539 and Cys544, Cd-B (740.2 ppm) to Cys525, Cys546, Cys553 and Cys556, and Cd-C (746.6 ppm) to Cys558 and Cys561. The other two ligands for Cd-C were missing in all spectra probably due to small ^3^J_Hβ-Cd_ value and could not be assigned.

### Structural Determination of MSL2 CXC Domain

The structure of MSL2 CXC domain was initially calculated in CYANA based solely on autoassigned NOE cross-peaks without incorporation of zinc ions. The resulting structures converged and showed a compact fold with Cys clustering at the center. The structure was further refined in CNS with additional TALOS-derived dihedral restraints, protein-zinc restraints deduced from ^113^Cd NMR and explicit water (see Materials and Methods). Inspection of structures calculated without the Zn-C restraints suggested that Cys539 and Cys553 are the remaining two ligands for Zn-C that could not be identified with ^113^Cd NMR. Other assignments of Zn-C ligands were not consistent with the existing structural restraints. The numbers and types of restraints used in the final structure calculation and the statistics for the 20 lowest energy structures are given in Table 1.

**Table 1:** Refinement statistics for the 20 lowest energy NMR structures of MSL2 CXC domain.

Restraint statistics	
Distance restraints	
Total NOE	1322
Intra-residue	407
Sequential (|*i* – *j*| = 1)	269
Medium-range (|*i* – *j*| ≤4)	111
Long-range (|*i* – *j*| ≥5)	160
Ambiguous	375
Total dihedral angle restraints	56
φ	28
ψ	28
**Structure statistics**	
Violations	
Max. dihedral angle violation (°)	4.88
Max. Distance constraint violation (Å)	0.151
Deviations from idealized geometry	
Bond lengths (Å)	0.0109±0.0004
Bond angles (°)	1.183±0.044
Impropers (°)	1.588±0.107
RMSD of all structures to mean (Å)	
Heavy (residues 523–529 and 538–565)	0.87±0.07
Backbone (residues 523–529 and 538–565)	0.38±0.06
Ramachandran plot analysis (all residues; residues 523–529 and 538–565)	
Most favored regions	77.2%; 80.7%
Additional allowed regions	21.3%; 19.3%
Generously allowed regions	0.7%; 0.0%
Disallowed regions	0.7%; 0.0%
WHAT_CHECK Z-scores (all residues; residues 523–529 and 538–565)	
2nd generation packing quality	−3.154; −1.963
Ramachandran plot appearance	−3.153; −2.028
chi-1/chi-2 rotamer normality	−1.725; −1.640
Backbone conformation	−5.322; −2.954

**Figure 4:**
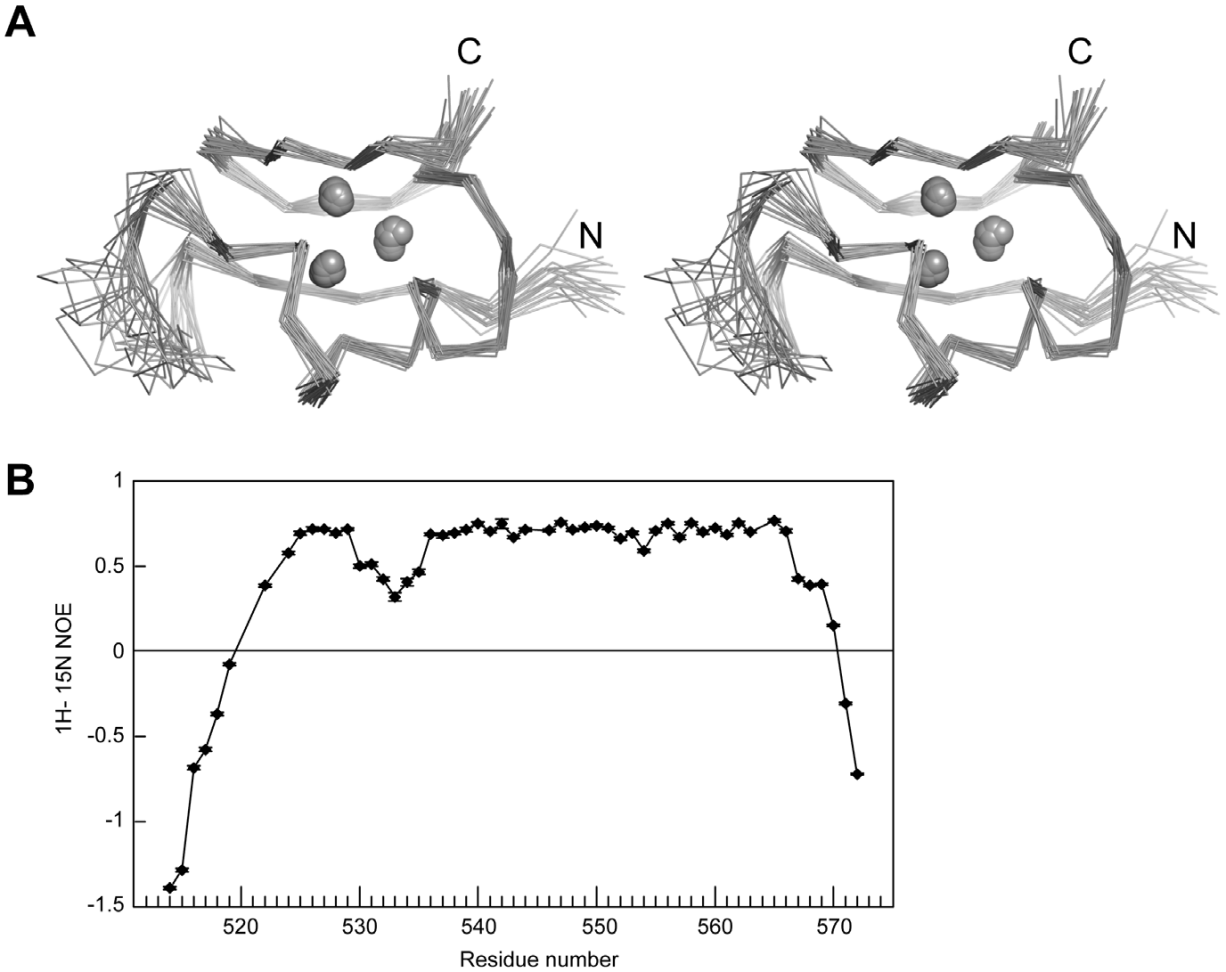
NMR structure and dynamics of MSL2 CXC domain. (A) Structural superposition of the 20 lowest energy NMR structures. The Cα traces of residues 521–566 and three zinc ions (spheres) are shown in cross-eye stereoview. (B) Steady-state ^1^H-^15^N heteronuclear NOE values are plotted as a function of residue number. The experiment was conducted for CXC-2, which contains residues 517–572 plus three extra N-terminal residues from the vector. No data were obtained for proline residues that lack amide proton. Error bars represent the experimental uncertainties estimated from the spectrum background noise.

The NMR structures are well defined with an average of 27 restraints per residue. The root mean square deviation (RMSD) values of the 20 best structures to the mean structure are 0.38 Å and 0.87 Å for backbone and heavy atoms in the structure core (residues 523–529 and 538–565), respectively (Fig. 4A). A few terminal residues and an internal loop (residues 530–537) are poorly defined in the structure because of a lack of long-range distance restraints. These residues are intrinsically dynamic on the ps-ns timescale, as evidenced by their reduced steady-state ^1^H-^15^N heteronuclear NOE values (Fig. 4B).

### The Solution Structure of MSL2 CXC Domain

**Figure 5:**
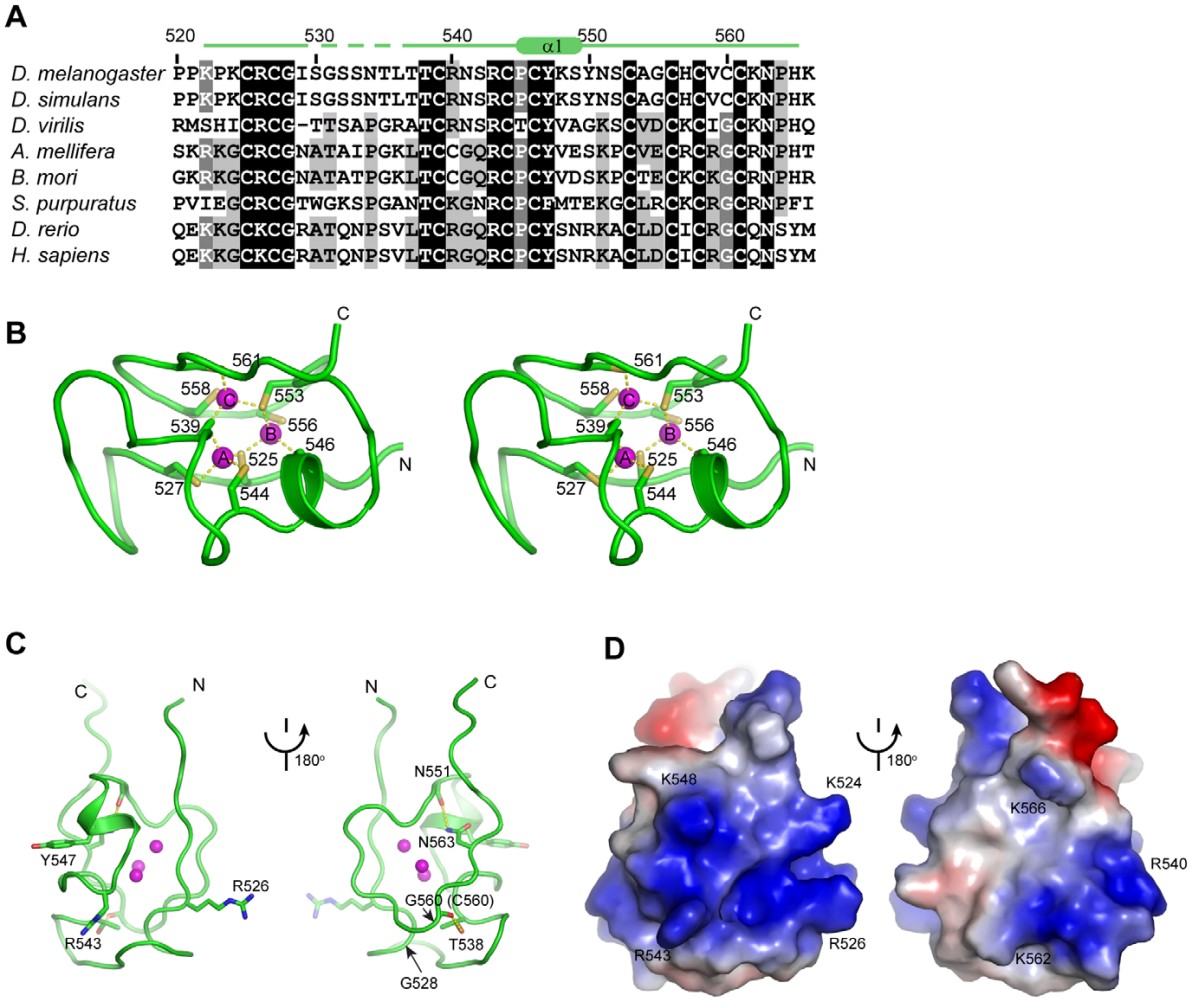
Important features of MSL2 CXC domain structure. (A) Sequence alignment of MSL2 homologs in the CXC domain. A total of 44 sequences were aligned and eight sequences are displayed. Residues conserved in 98%, 80% and 60% of 44 aligned sequences are shaded in black, gray and light gray, respectively. Similar amino acid groups are defined as follows: D and E; S and T; K and R; F, Y and W; L, I, V and M. The secondary structures are shown on the top with dotted line representing mobile regions. (B) Structure of the Zn_3_Cys_9_ cluster in cross-eye stereoview. The Zn-S coordination bonds are shown as dotted lines. (C) The highly conserved non-Cys residues of MSL2 CXC domain are displayed in sticks. Two views related by a 180° rotation are shown and dotted lines denote hydrogen bonds. (D) The electrostatic potential surface is colored from red to blue for negatively to positively charged regions. The structure is shown in the same two orientations as in (C).

The CXC domain adopts a small globular fold that encapsulates three triangularly arranged zinc ions (Fig. 5B). Among nine Cys ligands, six are singly coordinated, and three (Cys525, Cys539 and Cys553) simultaneously bind two zinc ions, such that each zinc ion is tetrahedrally coordinated to two terminal and two bridging Cys. The structure is composed of two loops that wrap around the Zn_3_Cys_9_ cluster in a right-handed manner. The N-terminal loop (residues 521–550) including a short α-helix (residues 546–550) harbors five Cys residues, whereas the C-terminal loop (residues 551–567) contains four Cys residues.

An intact Zn_3_Cys_9_ cluster appears to be important for the structure and function of CXC domains. The Ala double substitution of Cys544 and Cys546 of MSL2 was previously shown to delay development and reduce male fly viability [14]. The same double mutation impaired the DNA-binding ability of MSL2 and abolished its targeting to an HAS in *D. melanogaster* cells [28]. According to our structure, these two Cys residues are the ligand for Zn-A and Zn-B, respectively. The mutation likely disrupts the CXC domain structure, hence impairing its DNA-binding activity and in vivo function. In addition, Cys mutations in the CXC domain were shown to disrupt the function of TSO1 in flower development and cell division [34] and to impair the DNA-binding activity of human LIN54 [32].

Besides invariant Cys ligands, residues Arg526, Gly528, Thr538, Arg543, Try547 and Asn563 are also highly conserved among MSL2 homologs (Fig. 5A). These residues could be conserved for structural and/or functional reasons. In the structure, the side chains of Thr538 and Asn563 form hydrogen bonds with the carbonyl oxygen atoms of Gly560 and Asn551, respectively (Fig. 5C). These long range interactions apparently stabilize the small fold. The aromatic ring of Try747 is partially buried and Gly528 is located in a β-turn; these two residues probably also play a structural role. The Ala mutation of Try547 has been shown to disrupt the DNA binding and HAS targeting of MSL2 [28]. By contrast, the conserved Arg526 and Arg543 are solvent exposed and they together with other less conserved basic residues constitute a negatively charged surface patch (Fig. 5D). The two conserved arginine residues probably contribute to the function of CXC domain, such as DNA interaction.

**Figure 6:**
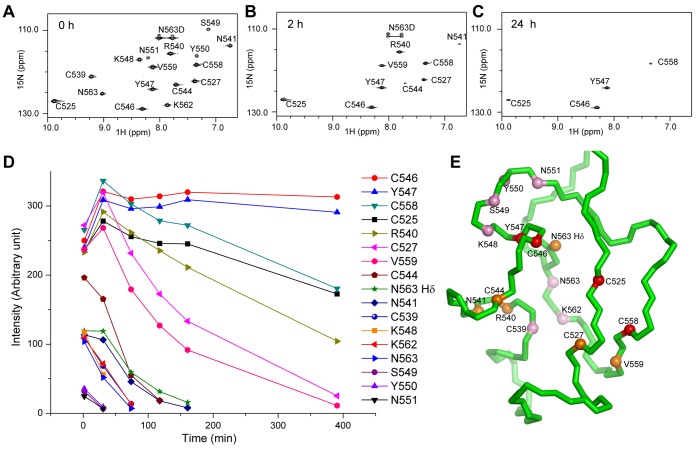
Amide proton exchange experiments. (A–C) ^1^H-^15^N HSQC spectra collected immediately (A), 2 h (B) or 24 h (C) after dissolving the lyophilized CXC-3 protein in ^2^H_2_O. The peaks are labeled and the side chain amide proton Hδ of N563 is labeled as N563D. (C) Intensity of amide proton peak as a function of exchange time. (D) Distribution of slow exchange amide protons in the CXC domain structure. The protected amide protons are shown as spheres on a backbone trace and are colored pink if present in the first recorded spectrum but not after 2 h, orange if present at 2 h but not after 24 h, and red if present after 24 h. The side chain of N563 is also displayed.

To validate the structure, we conducted proton-deuterium exchange experiment. The lyophilized CXC protein was dissolved in ^2^H_2_O and monitored with ^1^H-^15^N HSQC spectra for disappearance of amide proton. Seventeen amide protons were observed in the first recorded spectrum, ten persisted after 2 h and four remained after 24 h (Fig. 6A–D). According to our structure, these slow exchange amide protons are generally involved in hydrogen binding or are buried, and hence are protected from solvent exchange (Fig. 6E). In particular, the two side chain amide protons of Asn563, but not from other Asn or Gln, were protected, supporting that they are hydrogen bonded (Fig. 5C).

### Structural Similarity between CXC and Pre-SET Domains

Similar Zn_3_Cys_9_ clusters have been previously described in metallothioneins (MTs) and the SUV39 family of SET domain histone lysine methyltransferases (HKMTs). MTs are ubiquitous Cys-rich small proteins (6–7 kDa) with a high content of divalent metal ions such as Zn^2+^, Cd^2+^ and Cu^1+^ and are involved in metal homeostasis, detoxification and protection against reactive oxygen [37]. The mammalian MT II is composed of a β-domain that binds an M_3_Cys_9_ metal-thiolate cluster and an α-domain that binds an M_4_Cys_11_ cluster [38]. M_3_Cys_9_ clusters are also present in other families of MTs [39]–[41]. However, the structural fold and linear order of Cys ligands in MSL2 CXC domain are distinct from those in various types of structurally characterized MTs [38]–[41]. MTs and CXC domains are also unrelated in function.

Surprisingly, the CXC domain structure shows remarkable resemblance with structures of pre-SET motifs in the SUV39 family of HKMTs [42]–[44]. SET domain HKMTs have been classified into at least seven families, and the SET domains of SUV39, SET2 and EZ family proteins are preceded by a family-specific Cys-rich pre-SET motif [45].

**Figure 7:**
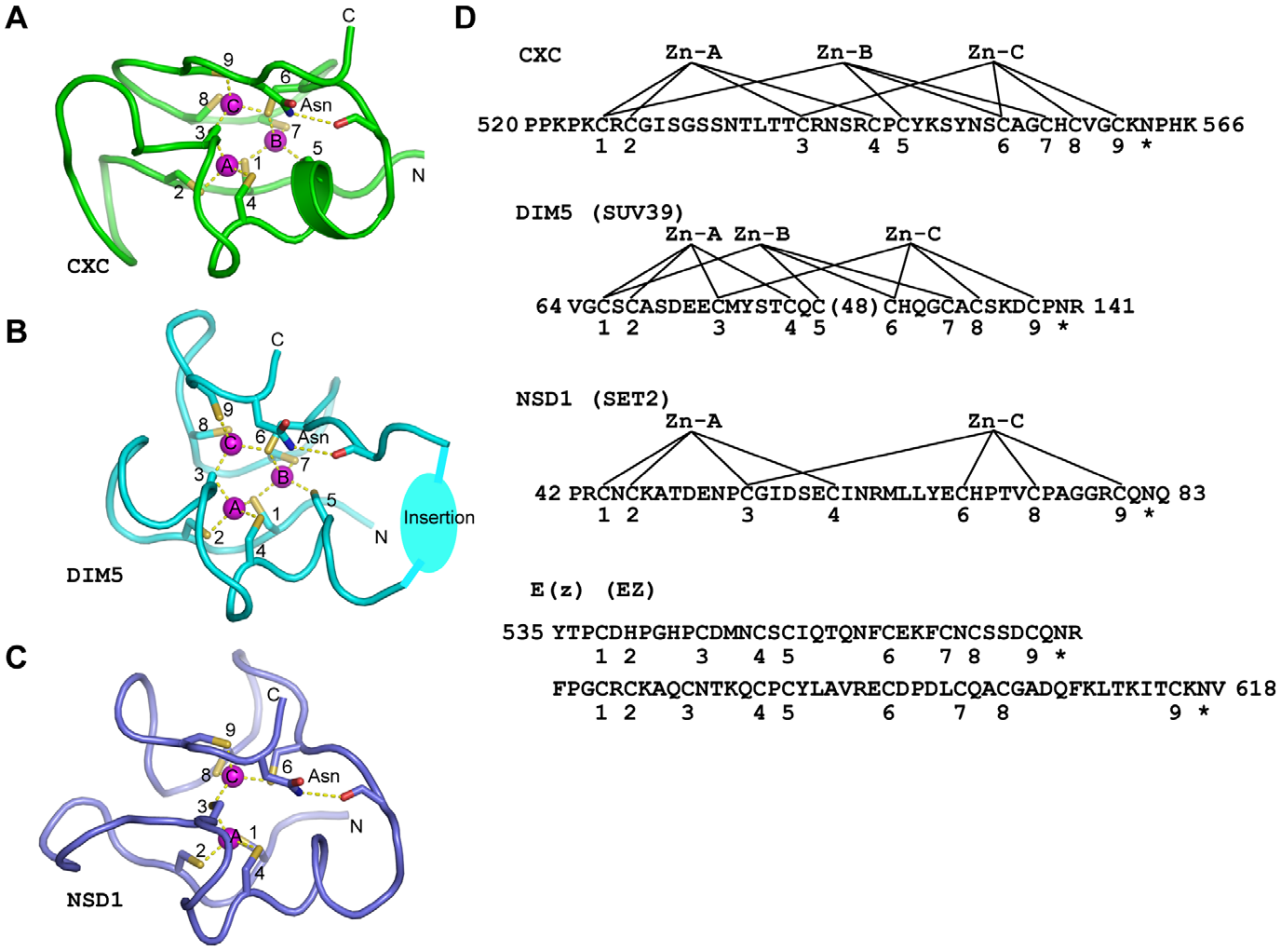
Structural similarity between CXC and pre-SET domains. (A) Structure of MSL2 CXC domain. The Cys ligands and the C-terminal invariant Asn residue are shown as sticks, zinc ions as purple spheres and Zn-S coordination bonds and hydrogen bonds as dotted lines. (B) Structure of the SUV39 family Dim5 pre-SET domain (PDB code 1ML9). The long insertion between Cys-5 and Cys-6 is shown schematically as an oval. (C) Structure of the SET2 family NSD1 pre-SET domain (PDB code 3OOI). The three structures of MSL2 CXC, Dim5 pre-SET and NSD1 pre-SET domains are aligned by their Zn-Cys clusters. (D) The primary sequences of MSL2 CXC domain and pre-SET motifs of *Neurospora crassa* Dim5, human NSD1 and *Drosophila melanogaster* E(z). The observed or predicted zinc ligands are numbered sequentially from 1 to 9. The invariant C-terminal Asn is marked with asterisk. The coordination patterns of zinc ions observed in these structures are shown. The starting and ending residues of each sequence are labeled with residue numbers. A 48-residue region is omitted in DIM5 pre-SET. The zinc ligands in NSD1 pre-SET are numbered according to those in the CXC domain.

Structures of SUV39 family HKMTs show that the pre-SET domain coordinates three zinc ions with nine invariant Cys residues [42]–[44]. Like the MSL2 CXC domain, the zinc-binding structure of SUV39 pre-SET domains is composed of two loops with five and four Cys residues (Fig. 7A,B). Importantly, the linear order of Cys ligands for each of three zinc ions are strictly conserved between MSL2 CXC and SUV39 pre-SET domains (Fig. 7D). The spacing of Cys normally varies within two residues except in two regions. First, the SUV39 pre-SET domains contain an insertion of 25–60 residues between Cys-5 and Cys-6 that interacts with the SET domain. Second, the segment between Cys-2 and Cys-3 is longer in MSL2 CXC domain (11 residues) than that in SUV39 pre-SET domains (2–5 residues).

The unexpected structural similarity of SUV39 pre-SET domains and the CXC domain led us to examine whether other pre-SET motifs are related to the CXC domain. Several crystal structures have been recently determined for SET2 family HKMTs ([46], [47] and PDB 3H6L). These structures show that the pre-SET motif of SET2 proteins, which is also known as associated with SET domain (AWS), contains a Zn_2_Cys_7_ cluster (Fig. 7C). Despite having a different ZnCys cluster, the zinc-binding structure of the SET2 pre-SET domain bears significant similarity to the SUV39 pre-SET and CXC structures. The SET2 pre-SET domain can be considered a CXC domain variant that has a similar binding mode for Zn-A and Zn-C but that loses binding of Zn-B because of the absence of Cys-5 and Cys-7 equivalents (Fig. 7D). Cys-3 still bridges Zn-A and Zn-C as in CXC and SUV39 pre-SET structures.

No structure is currently available for the EZ family HKMTs. The EZ pre-SET motif contains 17 invariant Cys residues within about an 80-residue region. Some sequence similarity has been noted between the CXC and EZ pre-SET domains [31], [33]–[35]. In fact, the name of “CXC domain” was originally coined to describe the pre-SET motif of EZ proteins [48] and later adopted to designate the Cys-rich regions in TSO1 and MSL2 proteins [31], [35]. A single CXC domain was previously identified in the EZ pre-SET, but it lacks an equivalent of Cys-7 [31]. In considering the structural restraints that nine ligands are required for binding three zinc ions, we revised the alignment and identified two CXC domains in EZ pre-SET (Fig. 7D). In this new alignment, the second ligand of the N-terminal CXC domain is His, which is invariant in EZ pre-SET, rather than Cys. Histidine has been shown to coordinate zinc clusters as a terminal ligand [49]. The tandem CXC domains of EZ pre-SET are immediately adjacent to each other in contrast with those found in tesmin/TSO1 proteins, which are separated by 40–60 residues.

We found that the second position C-terminal of Cys-9 is always occupied by an Asn residue in CXC and three types of pre-SET domains (Fig. 7D). In the crystal structures of SUV39 and SET2 family proteins, the equivalent Asn plays an important role in stabilizing the conformation of the C-terminal loop with its side chain amide nitrogen making a hydrogen bond to the carbonyl oxygen of the second residue N-terminal of Cys-6 (Fig. 7B, C). In the NMR structure of MSL2 CXC domain, the Asn side chain makes a similar interaction with the polypeptide backbone (Fig. 7A). The C-terminal Asn is also invariant in the CXC domains of tesmin/TSO1 proteins and the two reassigned CXC domains of EZ pre-SET. These findings indicate that the C-terminal Asn is a signature residue of the CXC superfamily.

In summary, we show that three types of pre-SET motifs are all related to the CXC domain. SUV39 pre-SET is a CXC domain with a large insertion, SET2 pre-SET is a variant CXC domain lacking one zinc ion and EZ pre-SET appears to contain tandem CXC domains. We can define the consensus sequence of CXC superfamily as CX[C/H]X_2–13_CX_4–7_CXCX_5–60_CX_2–4_CX_1–2_CX_2–15_CXN.

## Discussion

The MSL1 and MSL2 complex recognizes HAS in the X chromosome and mediates the first step of X-targeting by the DCC. The CXC domain is the only DNA-binding domain identified so far in the MSL1/MSL2 complex and contributes critically to the recognition of HAS by MSL2 in vivo [28]. We have determined the first structure of MSL2 CXC domain by NMR spectrometry, revealing a surprising Zn_3_Cys_9_-containing fold. The structure reveals the role of nine invariant Cys residues in coordinating three zinc ions. The strong sequence conservation suggests that the CXC domain of tesmin/TSO1 family proteins should adopt a similar structure, including a Zn_3_Cys_9_ cluster, as MSL2 CXC domain.

We have identified unexpected structural homology between CXC and pre-SET domains, suggesting that they share a common ancestor domain in evolution. This finding also expands the structural diversity and distribution of the CXC domain superfamily. These deviant CXC domains have large variations in Cys spacing, degenerate zinc binding ligands or His ligands and are thus difficult to recognize if no structure is available. The structural knowledge and derived consensus sequence of the CXC domain superfamily may allow for more deviant CXC domains to be identified.

No specific function other than a structural role has been assigned to pre-SET domains. DNA binding appears to be the primary function of CXC domains [28], [32], [33]. The homology with the CXC domain implicates that pre-SET domains may have a role in binding DNA, which could facilitate HKMT recognition of nucleosomal substrates.

Many DNA-binding domains, such as helix-turn-helixes, zinc fingers and leucine zippers, use an α-helix to contact DNA at the major groove. The CXC structure is distinct from previously characterized DNA-binding domains and apparently lacks such a DNA-binding α-helix. Determining the CXC domain complex structure with DNA will help to elucidate its likely distinct DNA-binding mode.

## Materials and Methods

### Protein Expression and Purification

The MSL2 cDNA was obtained from the Drosophila Genomics Resource Center. The coding sequence of the CXC domain corresponding to residues 517–572 was amplified by PCR and cloned into a pGEX-6p-1 vector (GE Healthcare). The mutation C560G was introduced by QuikChange site-directed mutagenesis (Stratagene), yielding the GST-fused CXC-2 construct. The coding sequence consisting of residues 520–570 and the C560G mutation was amplified from the CXC-2 plasmid and subcloned into an engineered pET28a vector, yielding the CXC-2 construct. The CXC-3 protein was expressed as fusion to an N-terminal His_6_-SMT3 tag. Single point mutations of R526A and R543A were introduced into CXC-3 by QuikChange. All constructs were confirmed by DNA sequencing.

*Escherichia coli* Rosetta(DE3) cells containing CXC expression vectors were grown at 37°C in LB broth. When the OD_600_ reached 0.8, the growth temperature was lowered to 16°C, and the culture media was supplemented with 40 µM ZnSO_4_ and 0.2 mM isopropyl-β-D-thiogalactopyranoside to induce protein expression. After additional growth for 16 h, the cells were harvested, suspended in buffer A consisting of 50 mM Tris-HCl (pH 8.0) and 250 mM NaCl, lysed by sonication and centrifuged.

The clarified lysate of GST-fused CXC-2 was loaded onto a GSTrap column. The GST-tag was cleaved on column by PreScission protease overnight. The released CXC-2 protein was washed with buffer A, diluted 3-fold with 25 mM HEPES-K (pH 7.6), loaded onto a heparin column (GE Healthcare) equilibrated in 25 mM HEPES-K (pH 7.6) and 80 mM KCl and eluted using a linear gradient of KCl. Proteins eluting at less than 300 mM KCl were pooled, concentrated with 3-kDa cutoff ultrafiltration devices (Amicon) and further purified with a Superdex 75 column in buffer 50 mM phosphate (pH 6.0).

The His_6_-SMT3-tagged CXC-3 protein was purified with a HisTrap column (GE Healthcare) and eluted with 500 mM imidazole in buffer A. The pooled factions were incubated with ULP1 for 1 h on ice to cleave the His_6_-SMT3 tag. After a threefold dilution in 25 mM HEPES-K (pH 7.6), the protein was loaded onto a Q column. The flow-through containing CXC-3 was further purified by heparin and gel filtration chromatography following the same procedure as for CXC-2. Protein concentrations were determined spectrophotometrically with a calculated molar extinction coefficient of 4470 M^−1^ cm^−1^ at 280 nm for both CXC-2 and CXC-3.

^15^N- or ^15^N/^13^C- labeled CXC proteins were prepared in M9 minimal medium with 1 g/L of (^15^NH_4_)_2_SO_4_ and, if needed, 2 g/L of ^13^C-glucose as the sole nitrogen and carbon sources, respectively (Cambridge Isotope Laboratories). For ^113^Cd labeling, ZnCl_2_ in M9 media was substituted with 10 nM ^113^Cd acetate. *E. coli* growth and protein yields were normal in ^113^Cd-containing media.

### Electrospray Ionization Mass Spectrometry

The CXC-3 protein was exchanged into 200 mM ammonium acetate and analyzed by electrospray ionization mass spectrometry with a Q-Star mass spectrometer (Applied Biosystems). To calculate the monoisotopic mass of a species, the mass-to-charge (m/z) ratio of its monoisotopic peak was multiplied by the charge, and the charge was then subtracted. The monoisotopic mass of CXC-3, which contains residues 520–570, mutation C560G and an extra N-terminal Ser from the vector, is 5638.48 Da. The exact mass of Zn^2+^ ion was calculated as molecular mass of ^64^Zn (63.929) minus 2, to compensate for two positive charges of Zn^2+^ ion.

### NMR Experiments

The NMR samples contained 0.5–1.5 mM CXC-2 or CXC-3 proteins, 50 mM potassium phosphate (pH 6.0), 0.01% (w/v) sodium 2,2-dimethylsilapentane-5-sulfonate (DSS) and 10% (v/v) ^2^H_2_O. NMR data were recorded at 298 K on a Bruker DMX600 spectrometer equipped with a triple resonance cryoprobe, unless indicated otherwise. Three-dimensional (3D) CBCA(CO)NH, HNCACB and HNCO spectra were collected to obtain sequence-specific backbone resonance assignments [50]. 3D HAHB(CO)NH, CC(CO)NH, CCH-TOCSY (mixing time τ_m_ 12 ms), HCCH-TOCSY (τ_m_ 12 ms), ^1^H-^15^N TOCSY-HSQC (τ_m_ 60 ms) and 2D ^1^H-^13^C HSQC spectra were collected for side-chain assignments. All spectra were processed with Felix (Accelrys Inc.) and analyzed with NMRViewJ [51]. The ^1^H chemical shifts were referenced to internal DSS, and the ^15^N and ^13^C chemical shifts were referenced indirectly. The ^1^H-^15^N steady-state heteronuclear NOE values were calculated from the ratios of peak intensities in a ^1^H-^15^N HSQC spectrum collected with a 3 s period of initial proton saturation to those in an unsaturated spectrum. The error of peak intensity was estimated from spectrum background noise and propagated into the error of ^1^H-^15^N heteronuclear NOE.

In hydrogen-deuterium exchange experiments, the CXC-3 protein originally prepared in 500 µl of 50 mM potassium phosphate (pH 6.0) was lyophilized and redissolved in 500 µl^ 2^H_2_O. A series of ^1^H-^15^N HSQC spectra were recorded 0 min, 30 min, 75 min, 120 min, 160 min, 390 min and 24 h after sample preparation.

### ^113^Cd NMR Experiments

1D ^113^Cd, 2D ^1^H-^113^Cd HSQC and 2D ^1^H-^113^Cd HMQC-TOCSY spectra were recorded on a Bruker 400 MHz NMR spectrometer equipped with a broadband double resonance probe. The delays for magnetization transfer (1/2J_H-Cd_) were set to 10, 16.7 and 25 ms. The resonances of ^113^Cd-loaded CXC were reassigned according to the assignments of Zn-loaded CXC and 3D ^1^H-^15^N TOCSY-HSQC and ^1^H-^15^N NOESY-HSQC spectra collected on ^113^Cd/^15^N-labeled CXC protein. The ^113^Cd chemical shifts are reported relative to external 1 M Cd(CH_3_COO)_2_, which serves as the 0 ppm reference.

### Structure Calculation

NOE-based distance restraints were derived from 3D ^1^H-^15^N NOESY-HSQC (τ_m_ 200 ms), 3D aliphatic ^1^H-^13^C NOESY-HSQC (τ_m_ 200 ms) and 3D aromatic ^1^H-^13^C NOESY-HSQC (τ_m_ 200 ms) spectra. Inter-proton distances were obtained with NMRViewJ using an exponential calibration from peak volumes and the upper limits of restraints were set to 2.2–6.0 Å. The CXC structure was initially calculated in CYANA solely from autoassigned NOE peaks [52]. More than 80% of NOE peaks were assigned this way, and the structure calculation converged with an RMSD of 1.12 Å for the backbone atoms of the 20 best structures.

The CYANA-generated model was further refined in CNS [53], incorporating additional dihedral angle and Zn restraints. The CYANA assignments of the NOE peaks were checked manually. Backbone dihedral angle restraints were derived from HN, Hα, Cα, Cβ, C' and N chemical shifts using TALOS+ [54]. The Zn tetrahedral coordination geometry was maintained by setting the bond length of Zn-Sγ(Cys) as 2.3 Å and the bond angle of Sγ(Cys)-Zn-Sγ(Cys) as 109.5 degree. The Zn restraints that can be assigned by ^113^Cd NMR experiments were incorporated first. Zn-C was assigned by ^113^Cd NMR to be ligated to Cys558 and Cys561. Inspection of the resulting structures suggested that Cys539 and Cys553 are the remaining two ligands for Zn-C. Incorporation of these two Zn-C restrains caused no NOE violations during the structure calculation, whereas coordination of Zn-C with different ligands caused violations around the Zn-cysteine cluster, indicating the correctness of the assignment.

100 structures were calculated, and the 50 lowest energy structures were further refined with electrostatic potentials and explicit water using CNS and RECOORDScript [55]. The 20 lowest energy structures were selected for final analysis using PROCHECK-NMR [56], MolMol [57] and WHAT_CHECK [58]. Structural figures were created with PyMOL [59].

### Accession Numbers

The NMR resonance assignments for CXC-3 have been deposited in the BioMagResBank with accession number 18514. The atomic coordinates and experimental NMR restraints for the MSL2 CXC domain have been deposited in the Protein Data Bank with accession code 2LUA.

## References

[1] Conrad T, Akhtar A (2011) Dosage compensation in Drosophila melanogaster: epigenetic fine-tuning of chromosome-wide transcription. Nat Rev Genet 13: 123–134. 10.1038/nrg3124 22251873

[2] Gelbart ME, Kuroda MI (2009) Drosophila dosage compensation: a complex voyage to the X chromosome. Development 136: 1399–1410. 19363150 10.1242/dev.029645 PMC2674252

[3] Straub T, Becker PB (2007) Dosage compensation: the beginning and end of generalization. Nat Rev Genet 8: 47–57. 17173057 10.1038/nrg2013

[4] Kadlec J, Hallacli E, Lipp M, Holz H, Sanchez-Weatherby J, et al (2011) Structural basis for MOF and MSL3 recruitment into the dosage compensation complex by MSL1. Nat Struct Mol Biol 18: 142–149. 21217699 10.1038/nsmb.1960

[5] Morales V, Straub T, Neumann MF, Mengus G, Akhtar A, et al (2004) Functional integration of the histone acetyltransferase MOF into the dosage compensation complex. EMBO J 23: 2258–2268. 15141166 10.1038/sj.emboj.7600235 PMC419912

[6] Scott MJ, Pan LL, Cleland SB, Knox AL, Heinrich J (2000) MSL1 plays a central role in assembly of the MSL complex, essential for dosage compensation in Drosophila. EMBO J 19: 144–155. 10619853 10.1093/emboj/19.1.144 PMC1171786

[7] Kelley RL, Wang J, Bell L, Kuroda MI (1997) Sex lethal controls dosage compensation in Drosophila by a non-splicing mechanism. Nature 387: 195–199. 9144292 10.1038/387195a0

[8] Bashaw GJ, Baker BS (1997) The regulation of the Drosophila msl-2 gene reveals a function for Sex-lethal in translational control. Cell 89: 789–798. 9182767 10.1016/s0092-8674(00)80262-7

[9] Gilfillan GD, Straub T, de Wit E, Greil F, Lamm R, et al (2006) Chromosome-wide gene-specific targeting of the Drosophila dosage compensation complex. Genes Dev 20: 858–870. 16547172 10.1101/gad.1399406 PMC1475731

[10] Alekseyenko AA, Larschan E, Lai WR, Park PJ, Kuroda MI (2006) High-resolution ChIP-chip analysis reveals that the Drosophila MSL complex selectively identifies active genes on the male X chromosome. Genes Dev 20: 848–857. 16547173 10.1101/gad.1400206 PMC1472287

[11] Larschan E, Bishop EP, Kharchenko PV, Core LJ, Lis JT, et al (2011) X chromosome dosage compensation via enhanced transcriptional elongation in Drosophila. Nature 471: 115–118. 21368835 10.1038/nature09757 PMC3076316

[12] Kelley RL, Meller VH, Gordadze PR, Roman G, Davis RL, et al (1999) Epigenetic spreading of the Drosophila dosage compensation complex from roX RNA genes into flanking chromatin. Cell 98: 513–522. 10481915 10.1016/s0092-8674(00)81979-0

[13] Sural TH, Peng S, Li B, Workman JL, Park PJ, et al (2008) The MSL3 chromodomain directs a key targeting step for dosage compensation of the Drosophila melanogaster X chromosome. Nat Struct Mol Biol 15: 1318–1325. 19029895 10.1038/nsmb.1520 PMC2636508

[14] Lyman LM, Copps K, Rastelli L, Kelley RL, Kuroda MI (1997) Drosophila male-specific lethal-2 protein: structure/function analysis and dependence on MSL-1 for chromosome association. Genetics 147: 1743–1753. 9409833 10.1093/genetics/147.4.1743 PMC1208343

[15] Gu W, Szauter P, Lucchesi JC (1998) Targeting of MOF, a putative histone acetyl transferase, to the X chromosome of Drosophila melanogaster. Dev Genet 22: 56–64. 9499580 10.1002/(SICI)1520-6408(1998)22:1<56::AID-DVG6>3.0.CO;2-6

[16] Kageyama Y, Mengus G, Gilfillan G, Kennedy HG, Stuckenholz C, et al (2001) Association and spreading of the Drosophila dosage compensation complex from a discrete roX1 chromatin entry site. EMBO J 20: 2236–2245. 11331589 10.1093/emboj/20.9.2236 PMC125240

[17] Park Y, Mengus G, Bai X, Kageyama Y, Meller VH, et al (2003) Sequence-specific targeting of Drosophila roX genes by the MSL dosage compensation complex. Mol Cell 11: 977–986. 12718883 10.1016/s1097-2765(03)00147-3

[18] Oh H, Bone JR, Kuroda MI (2004) Multiple classes of MSL binding sites target dosage compensation to the X chromosome of Drosophila. Curr Biol 14: 481–487. 15043812 10.1016/j.cub.2004.03.004

[19] Dahlsveen IK, Gilfillan GD, Shelest VI, Lamm R, Becker PB (2006) Targeting determinants of dosage compensation in Drosophila. PLoS Genet 2: e5. 16462942 10.1371/journal.pgen.0020005 PMC1359073

[20] Alekseyenko AA, Peng S, Larschan E, Gorchakov AA, Lee OK, et al (2008) A sequence motif within chromatin entry sites directs MSL establishment on the Drosophila X chromosome. Cell 134: 599–609. 18724933 10.1016/j.cell.2008.06.033 PMC2613042

[21] Straub T, Grimaud C, Gilfillan GD, Mitterweger A, Becker PB (2008) The chromosomal high-affinity binding sites for the Drosophila dosage compensation complex. PLoS Genet 4: e1000302. 19079572 10.1371/journal.pgen.1000302 PMC2586088

[22] Grimaud C, Becker PB (2009) The dosage compensation complex shapes the conformation of the X chromosome in Drosophila. Genes Dev 23: 2490–2495. 19884256 10.1101/gad.539509 PMC2779748

[23] Zhou S, Yang Y, Scott MJ, Pannuti A, Fehr KC, et al (1995) Male-specific lethal 2, a dosage compensation gene of Drosophila, undergoes sex-specific regulation and encodes a protein with a RING finger and a metallothionein-like cysteine cluster. EMBO J 14: 2884–2895. 7796814 10.1002/j.1460-2075.1995.tb07288.x PMC398407

[24] Kelley RL, Solovyeva I, Lyman LM, Richman R, Solovyev V, et al (1995) Expression of msl-2 causes assembly of dosage compensation regulators on the X chromosomes and female lethality in Drosophila. Cell 81: 867–877. 7781064 10.1016/0092-8674(95)90007-1

[25] Bashaw GJ, Baker BS (1995) The msl-2 dosage compensation gene of Drosophila encodes a putative DNA-binding protein whose expression is sex specifically regulated by Sex-lethal. Development 121: 3245–3258. 7588059 10.1242/dev.121.10.3245

[26] Meller VH, Gordadze PR, Park Y, Chu X, Stuckenholz C, et al (2000) Ordered assembly of roX RNAs into MSL complexes on the dosage-compensated X chromosome in Drosophila. Curr Biol 10: 136–143. 10679323 10.1016/s0960-9822(00)00311-0

[27] Oh H, Park Y, Kuroda MI (2003) Local spreading of MSL complexes from roX genes on the Drosophila X chromosome. Genes Dev 17: 1334–1339. 12782651 10.1101/gad.1082003 PMC196065

[28] Fauth T, Muller-Planitz F, Konig C, Straub T, Becker PB (2010) The DNA binding CXC domain of MSL2 is required for faithful targeting the Dosage Compensation Complex to the X chromosome. Nucleic Acids Res 38: 3209–3221. 20139418 10.1093/nar/gkq026 PMC2879509

[29] Copps K, Richman R, Lyman LM, Chang KA, Rampersad-Ammons J, et al (1998) Complex formation by the Drosophila MSL proteins: role of the MSL2 RING finger in protein complex assembly. EMBO J 17: 5409–5417. 9736618 10.1093/emboj/17.18.5409 PMC1170866

[30] Wu L, Zee BM, Wang Y, Garcia BA, Dou Y (2011) The RING finger protein MSL2 in the MOF complex is an E3 ubiquitin ligase for H2B K34 and is involved in crosstalk with H3 K4 and K79 methylation. Mol Cell 43: 132–144. 21726816 10.1016/j.molcel.2011.05.015 PMC4119175

[31] Marin I (2003) Evolution of chromatin-remodeling complexes: comparative genomics reveals the ancient origin of “novel” compensasome genes. J Mol Evol 56: 527–539. 12698291 10.1007/s00239-002-2422-1

[32] Schmit F, Cremer S, Gaubatz S (2009) LIN54 is an essential core subunit of the DREAM/LINC complex that binds to the cdc2 promoter in a sequence-specific manner. FEBS J 276: 5703–5716. 19725879 10.1111/j.1742-4658.2009.07261.x

[33] Cvitanich C, Pallisgaard N, Nielsen KA, Hansen AC, Larsen K, et al (2000) CPP1, a DNA-binding protein involved in the expression of a soybean leghemoglobin c3 gene. Proc Natl Acad Sci U S A 97: 8163–8168. 10859345 10.1073/pnas.090468497 PMC16687

[34] Song JY, Leung T, Ehler LK, Wang C, Liu Z (2000) Regulation of meristem organization and cell division by TSO1, an Arabidopsis gene with cysteine-rich repeats. Development 127: 2207–2217. 10769244 10.1242/dev.127.10.2207

[35] Hauser BA, He JQ, Park SO, Gasser CS (2000) TSO1 is a novel protein that modulates cytokinesis and cell expansion in Arabidopsis. Development 127: 2219–2226. 10769245 10.1242/dev.127.10.2219

[36] Vasak M (1998) Application of 113Cd NMR to metallothioneins. Biodegradation 9: 501–512. 10335586 10.1023/a:1008346231847

[37] Coyle P, Philcox JC, Carey LC, Rofe AM (2002) Metallothionein: the multipurpose protein. Cell Mol Life Sci 59: 627–647. 12022471 10.1007/s00018-002-8454-2 PMC11337511

[38] Braun W, Vasak M, Robbins AH, Stout CD, Wagner G, et al (1992) Comparison of the NMR solution structure and the x-ray crystal structure of rat metallothionein-2. Proc Natl Acad Sci U S A 89: 10124–10128. 1438200 10.1073/pnas.89.21.10124 PMC50290

[39] Munoz A, Forsterling FH, Shaw CF, 3rd, Petering DH (2002) Structure of the (113)Cd(3)beta domains from Homarus americanus metallothionein-1: hydrogen bonding and solvent accessibility of sulfur atoms. J Biol Inorg Chem 7: 713–724. 12203008 10.1007/s00775-002-0345-3

[40] Riek R, Precheur B, Wang Y, Mackay EA, Wider G, et al (1999) NMR structure of the sea urchin (Strongylocentrotus purpuratus) metallothionein MTA. J Mol Biol 291: 417–428. 10438629 10.1006/jmbi.1999.2967

[41] Narula SS, Brouwer M, Hua Y, Armitage IM (1995) Three-dimensional solution structure of Callinectes sapidus metallothionein-1 determined by homonuclear and heteronuclear magnetic resonance spectroscopy. Biochemistry 34: 620–631. 7819257 10.1021/bi00002a029

[42] Wu H, Min J, Lunin VV, Antoshenko T, Dombrovski L, et al (2010) Structural biology of human H3K9 methyltransferases. PLoS One 5: e8570. 20084102 10.1371/journal.pone.0008570 PMC2797608

[43] Zhang X, Tamaru H, Khan SI, Horton JR, Keefe LJ, et al (2002) Structure of the Neurospora SET domain protein DIM-5, a histone H3 lysine methyltransferase. Cell 111: 117–127. 12372305 10.1016/s0092-8674(02)00999-6 PMC2713760

[44] Min J, Zhang X, Cheng X, Grewal SI, Xu RM (2002) Structure of the SET domain histone lysine methyltransferase Clr4. Nat Struct Biol 9: 828–832. 12389037 10.1038/nsb860

[45] Dillon SC, Zhang X, Trievel RC, Cheng X (2005) The SET-domain protein superfamily: protein lysine methyltransferases. Genome Biol 6: 227. 16086857 10.1186/gb-2005-6-8-227 PMC1273623

[46] Qiao Q, Li Y, Chen Z, Wang M, Reinberg D, et al (2011) The structure of NSD1 reveals an autoregulatory mechanism underlying histone H3K36 methylation. J Biol Chem 286: 8361–8368. 21196496 10.1074/jbc.M110.204115 PMC3048720

[47] An S, Yeo KJ, Jeon YH, Song JJ (2011) Crystal structure of the human histone methyltransferase ASH1L catalytic domain and its implications for the regulatory mechanism. J Biol Chem 286: 8369–8374. 21239497 10.1074/jbc.M110.203380 PMC3048721

[48] Hobert O, Jallal B, Ullrich A (1996) Interaction of Vav with ENX-1, a putative transcriptional regulator of homeobox gene expression. Mol Cell Biol 16: 3066–3073. 8649418 10.1128/mcb.16.6.3066 PMC231301

[49] Blindauer CA, Harrison MD, Parkinson JA, Robinson AK, Cavet JS, et al (2001) A metallothionein containing a zinc finger within a four-metal cluster protects a bacterium from zinc toxicity. Proc Natl Acad Sci U S A 98: 9593–9598. 11493688 10.1073/pnas.171120098 PMC55497

[50] Ferentz AE, Wagner G (2000) NMR spectroscopy: a multifaceted approach to macromolecular structure. Q Rev Biophys 33: 29–65. 11075388 10.1017/s0033583500003589

[51] Johnson BA (2004) Using NMRView to visualize and analyze the NMR spectra of macromolecules. Methods Mol Biol 278: 313–352. 15318002 10.1385/1-59259-809-9:313

[52] Herrmann T, Guntert P, Wuthrich K (2002) Protein NMR structure determination with automated NOE assignment using the new software CANDID and the torsion angle dynamics algorithm DYANA. J Mol Biol 319: 209–227. 12051947 10.1016/s0022-2836(02)00241-3

[53] Brunger AT, Adams PD, Clore GM, DeLano WL, Gros P, et al (1998) Crystallography & NMR system: A new software suite for macromolecular structure determination. Acta Crystallogr D Biol Crystallogr 54: 905–921. 9757107 10.1107/s0907444998003254

[54] Shen Y, Delaglio F, Cornilescu G, Bax A (2009) TALOS+: a hybrid method for predicting protein backbone torsion angles from NMR chemical shifts. J Biomol NMR 44: 213–223. 19548092 10.1007/s10858-009-9333-z PMC2726990

[55] Nederveen AJ, Doreleijers JF, Vranken W, Miller Z, Spronk CA, et al (2005) RECOORD: a recalculated coordinate database of 500+ proteins from the PDB using restraints from the BioMagResBank. Proteins 59: 662–672. 15822098 10.1002/prot.20408

[56] Laskowski RA, Rullmannn JA, MacArthur MW, Kaptein R, Thornton JM (1996) AQUA and PROCHECK-NMR: programs for checking the quality of protein structures solved by NMR. J Biomol NMR 8: 477–486. 9008363 10.1007/BF00228148

[57] Koradi R, Billeter M, Wüthrich K (1996) MOLMOL: A program for display and analysis of macromolecular structures J Mol Graph. 14: 51–55. 10.1016/0263-7855(96)00009-4 8744573

[58] Hooft RW, Vriend G, Sander C, Abola EE (1996) Errors in protein structures. Nature 381: 272. 8692262 10.1038/381272a0

[59] DeLano WL (2002) The PyMOL user's manual. San Carlos, CA, USA: Delano Scientific.

